# Vehicle Age and Driver Assistance Technologies in Fatal Crashes Involving Teen and Middle-Aged Drivers

**DOI:** 10.1001/jamanetworkopen.2025.8942

**Published:** 2025-05-07

**Authors:** Fangda Zhang, Christopher R. M. Rundus, Enas Alshaikh, Corinne Peek-Asa, Jingzhen Yang

**Affiliations:** 1 Center for Injury Research and Policy at the Abigail Wexner Research Institute, Nationwide Children’s Hospital, Columbus, Ohio; 2 Office of Research Affairs, University of California at San Diego, San Diego; 3 Department of Pediatrics, The Ohio State University, Columbus,

## Abstract

**Importance:**

Motor vehicle crashes are the leading cause of death for US teens. Newer vehicles and driver assistance technologies show promise in reducing crashes and injury severities; however, research on the age and technologies of vehicles driven by teens involved in fatal crashes is limited.

**Objective:**

To examine the differences in vehicle age and driver assistance technologies between vehicles driven by teen and middle-aged drivers involved in fatal crashes and to investigate the associations among vehicle age, driver assistance technologies, and driver death in these crashes.

**Design, Setting, and Participants:**

This retrospective cohort study used fatal crash data (2016-2021) obtained from the Fatality Analysis Reporting System. Data analysis was restricted to passenger vehicles. Participants included teen drivers (15-18 years old) and middle-aged drivers (31-55 years old). All analyses were performed between December 1, 2023, and July 25, 2024.

**Exposures:**

Exposures include the vehicle age (≤5, 6-15, or >15 years) and the number of driver assistance technologies installed (0 to 4).

**Main Outcomes and Measures:**

The main outcome was whether the driver died in fatal crashes. Multivariable logistic regressions examined the associations between vehicle age (or driver assistance technologies installed) and driver death in fatal crashes, adjusting for driver sex, restraint use, and crash year.

**Results:**

Among 81 145 drivers (49 838 male [61.4%]) involved in fatal crashes, there were 9809 teen drivers (mean [SD] age, 17.2 [0.9] years) and 71 336 middle-aged drivers (mean [SD] age, 41.7 [7.3] years). A higher proportion of teen drivers were operating vehicles older than 15 years compared with middle-aged drivers (2706 drivers [27.6%] vs 16 239 drivers [22.8%]). Driving vehicles aged 6 to 15 years (adjusted risk ratio [aRR], 1.19; 95% CI, 1.17-1.22) or older than 15 years (aRR, 1.31; 95% CI, 1.28-1.34) was associated with significantly higher odds of driver death in fatal crashes compared with driving vehicles 5 years old or newer, independently of driver age. Additionally, each installed driver assistance technology was associated with a 6% reduction (aRR, 0.94; 95% CI, 0.90-0.98) in the risk of driver death in fatal crashes.

**Conclusions and Relevance:**

These findings suggest that older vehicles and those with fewer driver assistance technologies are associated with increased risk of driver death in fatal crashes; thus, teens should drive the safest vehicles available, not older family cars. The findings underscore the urgent need to ensure teens drive safer vehicles to protect their lives.

## Key Points

**Question:**

Are teens likely to operate older vehicles or vehicles with fewer driver assistance technologies, and are these factors associated with driver death in fatal crashes?

**Findings:**

This cohort study of 81 145 drivers found that in fatal crashes teen drivers were more likely than middle-aged drivers to operate vehicles older than 15 years or vehicles with fewer driver assistance technologies. Older vehicles and those with fewer driver assistance technologies were associated with increased driver fatality risk.

**Meaning:**

These findings underscore the importance of safe vehicle strategies for teens, including operating newer vehicles and vehicles with more driver assistance technologies.

## Teaser

This cohort study examines whether teens are likely to operate older vehicles or vehicles with fewer driver assistance technologies and whether these factors are associated with driver death in fatal crashes.

## Introduction

Motor vehicle crashes (MVCs) are the leading cause of death for teenagers in the US.^1,2,3,4^ Despite substantial decreases in crash rates due to various traffic safety efforts, teen drivers remain disproportionately represented in both MVC-related injuries and fatalities.^5,6,7,8,9^ Per mile driven, teens have nearly 4 times the crash rate relative to adults aged 20 years and older.^10^ Teenagers also have the highest rates of MVC fatalities of all ages.^5,11,12^ From 2012 to 2021, fatalities among teen drivers (aged 15-20 years) increased by 13%.^13^ In 2021 alone, 2116 teen drivers died in traffic crashes in the US.^13^ Teens’ overrepresentation in MVC-related injuries and fatalities is not only due to their age and inexperience but also to driver error stemming from poor decision-making and risky driving behaviors.^1,3,10,14,15,16,17,18,19^

One approach to addressing driver error is through technology. Advances in vehicle technologies offer a promising means to reduce teen driver crashes and crash-related injury severities through the use of vehicle-based intelligent driver support systems.^20^ Modern vehicles are increasingly equipped with driver assistance technologies designed to enhance safety by providing direct, real-time support to drivers.^21,22,23,24,25^ These technologies help mitigate driver errors and reduce crashes, ultimately aiming to prevent MVC-related deaths.^21,22,26,27,28,29,30,31^ However, teen drivers tend to lag behind adult drivers in utilizing these technologies.^32^

Evidence from previous research indicates that teens are more likely than adults to drive older model vehicles and those lacking key safety features.^33,34^ Since newer vehicles are generally safer,^27,33,35,36^ teen drivers may be at a distinct disadvantage compared with adult drivers on the road. They may also be less protected owing to having fewer advanced safety systems in their vehicles.^37,38,39,40^ Given the magnitude of teen driver problems and their increasing involvement in fatal crashes,^13^ it is crucial to thoroughly understand the vehicles that teen drivers are operating, especially during fatal crashes. The existing evidence, although relevant, is limited and often based on self-reported data or outdated crash data. This highlights the need for an up-to-date understanding of the vehicles driven by fatally injured teens to guide future efforts in improving vehicle safety for teen drivers.^32^

This study aimed to (1) compare the vehicle age and the number of driver assistance technologies installed between vehicles driven by teen and middle-aged drivers in fatal crashes, and (2) examine the associations between vehicle age (and number of driver assistance technologies) and driver death in fatal crashes. We hypothesized that teen drivers were more likely than middle-aged drivers to operate older vehicles and vehicles with fewer driver assistance technologies. We also hypothesized that operating an older vehicle or a vehicle equipped with fewer driver assistance technologies would be associated with an increased risk of driver death in fatal crashes, independently of the driver’s age.

## Methods

### Data

We retrospectively analyzed data from Fatality Analysis Reporting System (FARS), a comprehensive crash database widely recognized in traffic safety research for its detailed information on drivers, vehicles, and crash environments involved in US fatal crashes. Per FARS’s definition, a fatal crash is defined as a police-reported crash involving a motor vehicle in transport on a trafficway in which at least 1 person dies within 30 days of the crash.^41^

**Figure 1.**
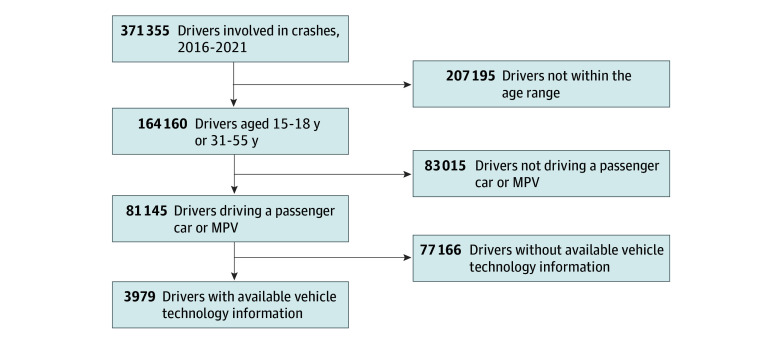
Flow Diagram Showing the Data Subsetting Process MPV indicates multipurpose passenger vehicle.

We combined several FARS subdatasets to obtain individual crash-level data with information about the vehicles and persons involved in fatal crashes. We then further refined the data by only including cases with individuals identified as drivers aged 15 to 18 years and 31 to 55 years, and vehicles categorized as passenger car or multipurpose passenger vehicle. Figure 1 is a flow diagram outlining the step-by-step data processing for the study. The selected data years spanned from 2016 to 2021, as FARS began recording the vehicle safety features of interest (eg, adaptive cruise control [ACC]) in 2016. We focused on driver fatalities as the outcome to investigate whether giving teens older family cars was associated with an increased risk of fatalities. This study was deemed exempt from institutional review board approval at our institution, and followed Strengthening the Reporting of Observational Studies in Epidemiology (STROBE) reporting guidelines for observational studies.^42^ Consent was not needed because the data are publicly available and anonymous, in accordance with 45 CFR §46. Since our analyses utilized the FARS dataset, which exclusively covers fatal crashes, we will not specify this context in every instance throughout the remaining sections.

### Outcome Variables and Measures

#### Driver Fatality

This variable indicates whether the driver died. In the original FARS dataset, drivers with a fatal injury were coded as 4, and unknown or not reported results were coded as 9 for years 2016 and later. We categorized drivers with code 4 as died, code 9 as unknown or not reported, and all other codes as not died.

#### Exposure Variables: Driver Age Group and Vehicle Age

The driver age group variable was categorized into 2 groups: teen drivers (aged 15-18 years^43^) and middle-aged drivers (aged 31-55 years). Vehicle age was calculated by subtracting the year of the crash by the vehicle’s model year.^38^ It was subsequently classified into 3 groups (ie, ≤5 years, 6-15 years, and >15 years). The vehicle age cutoff points were determined on the basis of previous work.^35,36^

#### Driver Assistance Technology

This was measured as the number of driver assistance technologies installed in a vehicle. We included the following technologies: (1) ACC; (2) forward collision prevention (FCP); (3) lane and side assist (LSA); and (4) lighting technologies. Only vehicles with reported information about the technologies were included (all vehicles with a record status of the technology of interest were 5 years old or newer).

For ACC, if a vehicle was reported in FARS to have standard ACC, we then scored it as 1; otherwise, we scored it as 0, meaning ACC was not available. For the other 3 technologies, each consisting of several safety systems, we scored each of them as 1 if at least 1 corresponding system was available or 0 if none were available. We then summarized the number of driver assistance technologies for a given vehicle with a score ranging from 0 to 4. Other variables, including driver sex, crash year, and driver seatbelt restraint use, were included and adjusted in the later regression analysis as covariates owing to their potential association with crashes and crash injury severity.^18,33,37,44,45^

### Statistical Analysis

We used χ^2^ tests to compare participants’ demographics, the proportion of drivers who died, vehicle age, and the presence and the number of driver assistance technologies between driver age groups over the studied period. Multivariable logistic regressions were used to assess whether operating older vehicles was associated with whether drivers died in fatal crashes. A subgroup analysis based on 3979 vehicles with recorded driver assistance technology information was conducted to test whether operating vehicles equipped with fewer driver assistance technologies was associated with driver fatalities. We tested the interaction effects between vehicle age and driver age group, as well as the interaction effects between number of driver assistance technologies and driver age group. No statistically significant interactions were found, so the interaction term was not included in either model. Both models adjusted for driver sex, age, restraint use, and crash year. Furthermore, we compared the vehicles driven by teen and middle-aged drivers who died in fatal crashes by assessing the differences in proportions, along with 95% CIs, between the 2 age groups, using marginal risk. All analyses were performed between December 1, 2023, and July 25, 2024, using SAS statistical software version 9.4 (SAS Institute). The statistical significance was set at the α = .05 level.

## Results

### Participants and Vehicle Characteristics

Of the 81 145 drivers included, 49 838 (61.4%) were male, 9809 (12.1%) were teens (aged 15-18), and 33 331 (41.1%) died in the crashes (Table 1). The mean (SD) age of teens was 17.2 (0.9) years, and that of middle-aged drivers was 41.7 (7.3) years. The proportion of teen drivers who died in fatal crashes was lower than that of middle-aged drivers (3730 teen drivers [38.0%] vs 29 601 middle-aged drivers [41.5%]). At the time of the crash, 22.7% of teen drivers (2230 drivers) were not using any type of restraint, compared with 23.6% of middle-aged drivers (16 811 drivers).

**Table 1.** Driver Demographics, Vehicle Age, and Driver Assistance Technologies by Driver Age Group

Characteristic	Drivers, No. (%)			*P* value^a^
	All drivers (N = 81 145)	Teen drivers (n = 9809)	Middle-aged drivers (n = 71 336)	
Year^b^				
2016	12 947 (16.0)	1659 (16.9)	11 288 (15.8)	<.001
2017	13 298 (16.4)	1682 (17.1)	11 616 (16.3)	
2018	12 889 (15.9)	1526 (15.6)	11 363 (15.9)	
2019	12 664 (15.6)	1411 (14.4)	11 253 (15.8)	
2020	13 638 (16.8)	1628 (16.6)	12 010 (16.8)	
2021	15 709 (19.4)	1903 (19.4)	13 806 (19.4)	
Whether drivers died				
Yes	33 331 (41.1)	3730 (38.0)	29 601 (41.5)	<.001^c^
No	47 178 (58.1)	6005 (61.2)	41 173 (57.7)	
Not reported or unknown	636 (0.8)	74 (0.8)	562 (0.8)	
Driver sex				
Female	31 245 (38.5)	3693 (37.6)	27 552 (38.6)	.07^c^
Male	49 838 (61.4)	6106 (62.2)	43 732 (61.3)	
Not reported or unknown	62 (0.1)	10 (0.2)	52 (0.1)	
Restraint use				
Shoulder and/or lap belt used	54 075 (66.6)	6553 (66.8)	47 522 (66.6)	.03^c^
Other types of restraint used	1197 (1.5)	168 (1.7)	1029 (1.4)	
None used	19 041 (23.5)	2230 (22.7)	16 811 (23.6)	
Not reported or unknown	6832 (8.4)	858 (8.8)	5974 (8.4)	
Vehicle age, y				
≤5	22 593 (27.8)	1780 (18.1)	20 813 (29.2)	<.001
6-15	39 607 (48.8)	5323 (54.3)	34 284 (48.1)	
>15	18 945 (23.3)	2706 (27.6)	16 239 (22.8)	
Driver assistance technologies (n = 3979)^d^				
ACC				
1	1484 (47.4)	134 (52.3)	1512 (52.6)	.94
0	1646 (52.6)	122 (47.7)	1362 (47.4)	
Forward collision prevention				
1	1771 (44.5)	135 (43.3)	1636 (44.6)	.65
0	2208 (55.5)	177 (56.7)	2031 (55.4)	
Lane and side assist				
1	1625 (40.8)	98 (31.4)	1527 (41.6)	<.001
0	2354 (59.5)	214 (68.6)	2140 (58.4)	
Lighting technologies				
1	2373 (59.6)	181 (58.0)	2192 (59.8)	.54
0	1606 (40.4)	131 (42.0)	1475 (40.2)	
No. of driver assistance technologies installed^e^				
0	504 (12.7)	43 (13.8)	461 (12.6)	.19
1	1126 (28.3)	90 (28.9)	1036 (28.2)	
2	1060 (26.6)	95 (30.4)	965 (26.3)	
3	987 (24.8)	68 (21.8)	919 (25.1)	
4	302 (7.6)	16 (5.1)	286 (7.8)	

Abbreviation: ACC, adaptive cruise control.

^a^ *P* value was based on χ^2^ test.

^b^ Year was treated as continuous in later analyses.

^c^ Test was performed excluding the not reported or unknown cases.

^d^ Data about vehicle driver assistance technology equipment were based on a subgroup analysis of the entire dataset in which at least 1 studied driver assistance technology had a record from Fatality Analysis Reporting System (n = 3979). For ACC, if a vehicle was reported to have standard ACC, it was scored as 1; otherwise, it was scored as 0, meaning ACC was not available. For the other 3 technologies, each consisting of several safety systems, each was scored as 1 if at least 1 corresponding system was available or 0 if none were available.

^e^ No. reflects the sum of the studied driver assistance technologies installed in a vehicle.

A significantly higher proportion of teens were driving vehicles older than 15 years compared with middle-aged drivers (2706 teen drivers [27.6%] vs 16 239 middle-aged drivers [22.8%]). Overall, fewer driver assistance technologies were installed in vehicles driven by teens compared with those driven by middle-aged drivers, although most comparisons were not statistically significant. Only LSA was significantly less often implemented in vehicles driven by teens compared with those driven by middle-aged drivers (98 teen drivers [31.4%] vs 1527 middle-aged drivers [41.6%]) (Table 1).

### Age and Technologies of Vehicles Driven by Teen and Middle-Aged Drivers

**Figure 2.**
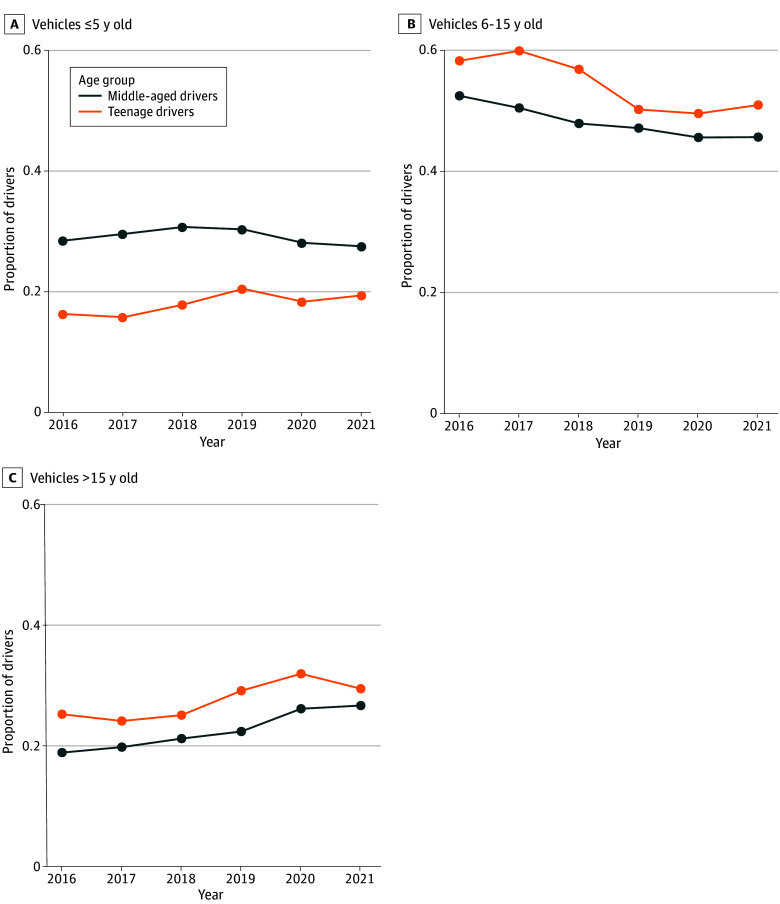
Proportion of Drivers by Vehicle Age and Driver Age Group at the Time of Fatal Crashes, 2016-2021 Graphs show data for vehicles that are no more than 5 years old (A), 6 to 15 years old (B), and older than 15 years (C).

From 2016 to 2021, the proportion of teen drivers operating older vehicles consistently remained higher compared with middle-aged drivers (Figure 2). During the same period, the proportion of vehicles equipped with any of the 4 driver assistance technologies increased in both age groups, with teen drivers consistently having a lower proportion driving vehicles equipped with the technologies. However, when analyzing vehicles 5 years or newer, the proportions of teen and middle-aged drivers operating vehicles equipped with driver assistance technologies were similar, except for LSA, where teens had lower proportion than middle-aged drivers (eFigure in Supplement 1).

### Age and Technologies of Vehicles Driven, by Driver’s Death

Vehicle age was associated with an increased risk of driver fatalities in fatal crashes for drivers in both age groups. Compared with drivers of vehicles 5 years old or less, driving a vehicle 6 to 15 years old was associated with 1.19 times (adjusted risk ratio [aRR], 1.19; 95% CI, 1.17-1.22) the risk of driver death, and driving a vehicle older than 15 years was associated with 1.31 times (aRR, 1.31; 95% CI, 1.28-1.34) the risk of driver death (Table 2). When examining the vehicles driven by drivers who died in fatal crashes, a significantly higher proportion of teen drivers were found to operate vehicles older than 15 years compared with middle-aged drivers, with a marginal risk difference of 2.82 (95% CI, 1.21-4.43) (Table 3). Results from the subgroup analysis on the vehicles with recorded technology information indicate that each additional driver assistance technology installed was associated with a 6% reduction in the risk of driver death in fatal crashes (aRR, 0.94; 95% CI, 0.90-0.98), independently of the driver’s age (Table 2).

**Table 2.** Associations Between Vehicle Age, Driver Assistance Technologies of Vehicles and Drivers Who Died and Did Not Die During Fatal Crashes

Variable and level	RR (95% CI)	
	Unadjusted	Adjusted
Vehicle age (n = 81 145)		
Vehicle age		
6-15 y vs ≤ 5 y	1.40 (1.37-1.43)	1.19 (1.17-1.22)
>15 y vs ≤ 5 y	1.88 (1.83-1.92)	1.31 (1.28-1.34)
Driver age group, teen drivers vs middle-aged drivers	0.92 (0.89-0.94)	0.88 (0.86-0.90)
Driver sex, male vs female	1.23 (1.21-1.26)	1.03 (1.02-1.05)
Restraint use		
Shoulder and/or lap belt used vs none used	0.35 (0.34-0.35)	0.37 (0.36-0.37)
Other types of restraint used vs none used	0.50 (0.47-0.54)	0.53 (0.50-0.57)
Year^a^	1.01 (1.00-1.02)	0.99 (0.99-1.00)
Driver assistance technologies (n = 3979)^b^		
No. of safety features equipped	0.89 (0.85-0.93)	0.94 (0.90-0.98)
Driver age group, teen drivers vs middle-aged drivers	0.90 (0.74-1.10)	0.76 (0.62-0.93)
Driver sex, male vs female	1.51 (1.35-1.66)	1.29 (1.16-1.44)
Restraint use		
Shoulder and/or lap belt used vs none used	0.29 (0.26-0.32)	0.31 (0.28-0.34)
Other types of restraint used vs none used	0.37 (0.24-0.56)	0.39 (0.26-0.59)
Year^a^	1.07 (1.02-1.12)	1.02 (0.98-1.06)

Abbreviation: RR, risk ratio.

^a^ Year was treated as continuous in the model; it was not significant when treated as categorical.

^b^ Data about vehicle driver assistance technology equipment were based on a subgroup analysis of the entire dataset in which at least 1 studied driver assistance technology had a record from Fatality Analysis Reporting System (n = 3979).

**Table 3.** Age of Vehicles Driven by Teen and Middle-Aged Drivers Who Died During Fatal Crashes

Vehicle age, y	Vehicles, No. (%)		Marginal risk difference (95% CI)
	Teen drivers (n = 3730)	Middle-aged drivers (n = 26 601)	
≤5	491 (13.16)	6124 (20.69)	−7.53 (−8.70 to −6.35)
6-15	1977 (53.00)	14297 (48.30)	4.70 (3.00 to 6.40)
>15	1262 (33.83)	9180 (31.01)	2.82 (1.21 to 4.43)

## Discussion

This cohort study examined the differences in vehicle age and driver assistance technologies between vehicles driven by teen and middle-aged drivers involved in fatal crashes, as well as the associations between these vehicle characteristics and driver fatalities using fatal crash data from FARS. The main findings indicate that teen drivers (15-18 years) were more likely than middle-aged drivers (31-55 years) to drive vehicles older than 15 years and vehicles with fewer driver assistance technologies at the time of fatal crashes. Furthermore, older vehicles and vehicles with fewer driver assistance technologies were associated with an elevated death risk for drivers involved in fatal crashes; these associations were observed for drivers in both age groups. Our results confirm previous study findings that fatality risks increase with vehicle age,^46^ including those based on FARS data from earlier years.^32,44^ Our findings also add to current literature that vehicles with more driver assistance technologies are associated with reduced risk of driver death in fatal crashes. These findings highlight the importance of operating newer vehicles and vehicles equipped with more driver assistance technologies.

Our results reveal that 27.6% of teen drivers were operating vehicles older than 15 years at the time of the fatal crash, significantly higher than 22.8% of middle-aged drivers. This is concerning because older vehicles often lack the critical safety features that could help prevent a crash or protect occupants if a crash occurs.^35,36^ In addition, older vehicles are more likely to malfunction due to degraded components like brakes and tires, less-robust design standards, and increased mechanical issues, which elevate the risk of crashes and crash fatalities.^35,36^ Currently, some states in the US mandate periodic safety inspections for all registered vehicles.^47^ Empirical evidence suggests that more frequent inspections can reduce vehicle defects, which contribute to crashes.^48,49^ Implementing such regulations nationwide for vehicles older than 15 years could therefore help ensure their safety.

Since parents often control what vehicles their teens drive,^34^ their choices greatly affect the driving safety of their teens and other road users.^32^ Parents commonly pass their old vehicles to their teens, who are still learning basic driving skills. This practice could increase teens’ vulnerability to vehicle malfunctions, making their driving less safe. Our findings, along with those from other studies,^33,36,37,38,39^ call for pediatricians and other health care practitioners to educate parents and teens about the risks of driving older vehicles. Parents should be advised to prioritize safety features when choosing the first car for their teens, ensuring it is newer and safer, given the increasing involvement of teen drivers in MVCs and MVC-related fatalities.^13,32^

Our study found that vehicles equipped with more driver assistance technologies were associated with a lower death risk for drivers involved in fatal crashes. Various driver assistance technologies are currently available to improve driving safety,^21,50,51^ including those that prevent crashes (eg, automatic emergency braking), enhance awareness (eg, blind spot monitoring), improve control (eg, stability and traction control), and reduce human error (eg, driver mistake compensation).^21,22,26,27,28,29^ However, many of these technologies are only available or required for newer vehicles. For example, backup cameras have been mandated to be installed in new cars sold in the US beginning in 2018.^52^ Most existing research examining driver assistance technologies has focused on the effect of individual technologies. Jermakian^50^ and Seacrist et al^51^ investigated crash avoidance technologies like automatic emergency braking and found them effective in mitigating crash severity, even if the crash was not entirely avoided. Unlike previous work, our study focused on the number of driver assistance technologies of the vehicles driven by drivers involved in fatal crashes. Additional research is needed to explore the associations between driver assistance technologies and the risk of crashes and crash-related injury severities among teen drivers.

Our findings provide insights into the vehicle characteristics (vehicle age and driver assistance technologies) of teen drivers involved in fatal crashes. These findings have important implications for pediatricians and other health care practitioners, who often play a crucial role in advising families on various health topics and who can be valuable partners in enhancing teen driving safety. On the basis of our findings, we propose several recommendations.

First, with regard to vehicle safety, teens should drive the safest vehicles available, not older family cars.^33,34^ Pediatricians and other health care practitioners should advise parents to prioritize safety features when choosing the first car for their teens and avoid vehicles older than 15 years,^35^ especially during the initial months of unsupervised driving, which carries teen drivers’ highest crash risk.^53,54,55^ Parents can refer to the Insurance Institute for Highway Safety for a list of affordable, safe vehicles for teens.^56^ If a newer vehicle is not an option, more-frequent maintenance should be encouraged to improve the vehicle’s safety.^35^

Second, with regard to newer technologies, pediatricians and other health care practitioners should educate families about the benefits of newer vehicle technologies, such as crash avoidance features and teen-specific technologies, which can significantly reduce crashes and related injuries. They should recommend vehicles with more driver assistance technologies for teens whenever possible.

Third, for safe driving habits, pediatricians and other health care practitioners should address other aspects of teen driving safety beyond vehicle selection. They should educate parents and teens about the danger of risky driving behaviors and promote safe driving habits, such as seat belt use, safe nighttime driving, limiting teen passengers, avoiding distractions, and adhering to state Graduated Driver Licensing requirements.

### Limitations

This study has several limitations. First, we relied solely on fatal crash data from FARS; thus, our study could not determine the risk of fatality, nor should our results be interpreted directionally. Second, the relatively small number of vehicles equipped with the technologies limited our ability to further examine the association between each driver assistance technology and driver death in fatal crashes. For example, very few drivers in this study were driving vehicles equipped with FCP fully or partially, despite FCP being a promising technology for mitigating crash severity and fatalities. Third, the driver assistance technologies analyzed were not comprehensive, and vehicle data recorded in FARS might not be accurate due to missing information. It is also possible that certain driver assistance technologies were installed but not activated at the time of the fatal crashes. Fourth, although many other factors, including driver behaviors (driving under influence), crash type (single vs multiple vehicle crash), vehicle model and year, and road conditions, could also contribute to driver fatalities,^9,37,57^ they were not examined in this study as they were not our primary focus.

## Conclusions

Teen driver fatalities are a serious public health concern. Given teen drivers’ high crash rates compared with other age groups and their increasing involvement in fatal crashes, it is crucial for teen drivers to operate the safest vehicles available rather than older family hand-me-downs. This study leveraged recent national fatal crash data and examined the vehicle age and driver assistance technologies of vehicles driven by teen and middle-aged drivers, and their associations with driver fatalities during fatal crashes. Our findings indicate that teen drivers were more likely to drive older vehicles and vehicles with fewer driver assistance technologies during fatal crashes, and that such vehicles were associated with a higher risk of death for drivers involved in fatal crashes regardless of drivers’ age. Our results underscore the need and importance to implement safe vehicle strategies and ensure teens drive safer cars. Further efforts from parents, health care practitioners, and safety professionals are needed to ensure the driving safety of all road users.

## Notes

Supplement 1. **eFigure.** Proportion of drivers driving vehicles 5 years or newer and with a) standard ACC; b) full or partial FCP; c) full or partial LSA); d) full or partial LT at the time of fatal crashes by respective driver age group, 2016-2021

Supplement 2.
Data Sharing Statement
