# Supplementary material for: Vehicle Age and Driver Assistance Technologies in Fatal Crashes Involving Teen and Middle-Aged Drivers
Source: JAMA Netw Open. 2025 May 7;8(5):e258942. doi: 10.1001/jamanetworkopen.2025.8942 (PMC12059974; doi:10.1001/jamanetworkopen.2025.8942)
Supplement: Supplement 1. — eFigure. Proportion of drivers driving vehicles 5 years or newer and with a) standard ACC; b) full or partial FCP; c) full or partial LSA); d) full or partial LT at the time of fatal crashes by respective driver age group, 2016-2021 [file jamanetwopen-e258942-s001.pdf]

Supplemental Online Content

Zhang F, Rundus CRM, Alshaikh E, Peek-Asa C, Yang J. Vehicle age and driver assistance technologies in fatal crashes involving teen and middle-aged drivers. *JAMA Netw Open*. 2025;8(5):e258942. doi:10.1001/jamanetworkopen.2025.8942

eFigure. Proportion of drivers driving vehicles 5 years or newer and with a) standard ACC; b) full or partial FCP; c) full or partial LSA); d) full or partial LT at the time of fatal crashes by respective driver age group, 2016-2021

This supplemental material has been provided by the authors to give readers additional information about their work.

© 2025 Zhang F et al. *JAMA Network Open*.

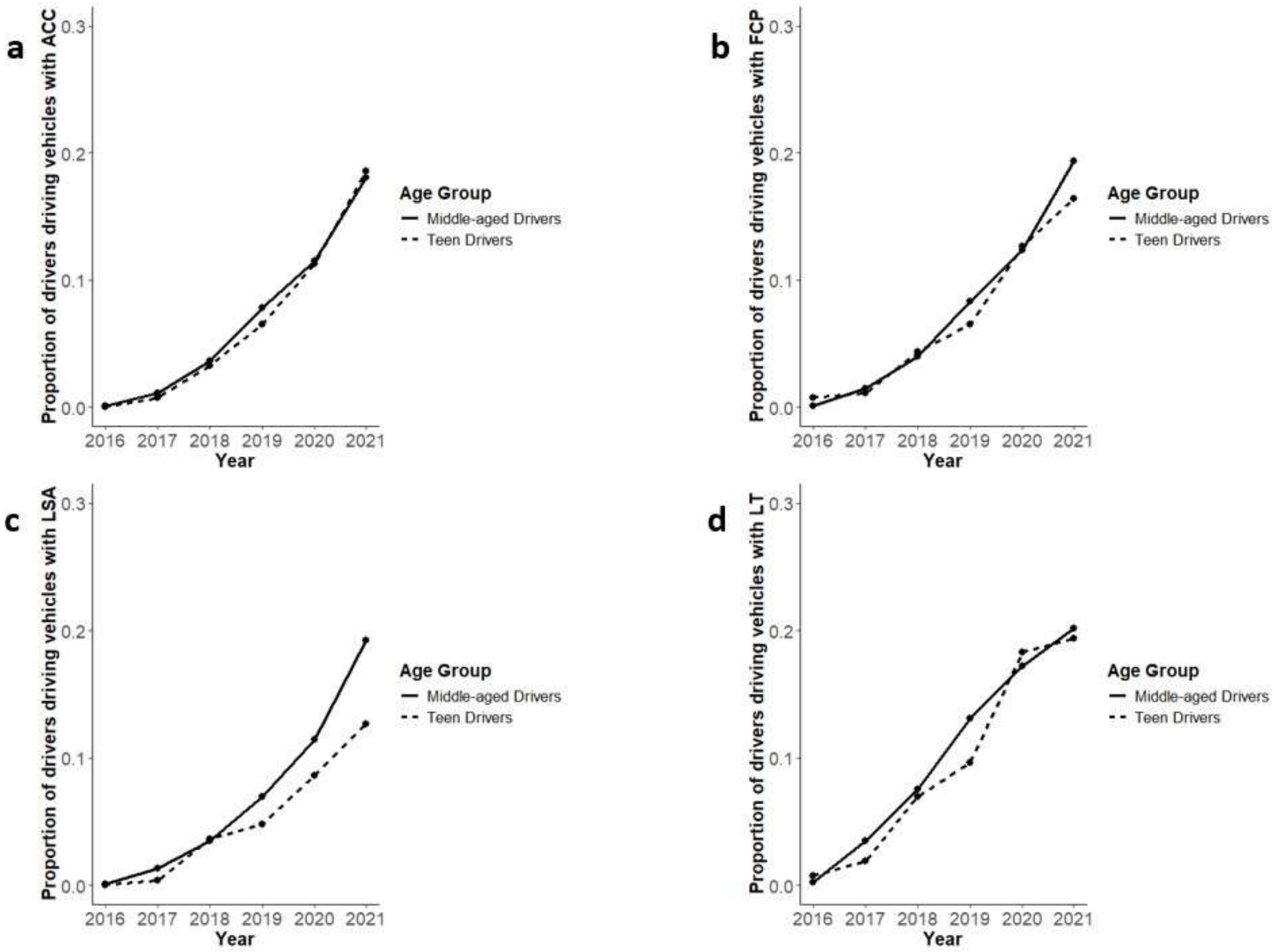

eFigure. Proportion of drivers driving vehicles 5 years or newer and with a) standard ACC; b) full or partial FCP; c) full or partial LSA); d) full or partial LT at the time of fatal crashes by respective driver age group, 2016-2021

© 2025 Zhang F et al. *JAMA Network Open*.
