# Supplementary material for: Vehicle Age and Driver Assistance Technologies in Fatal Crashes Involving Teen and Middle-Aged Drivers
Source: JAMA Netw Open. 2025 May 7;8(5):e258942. doi: 10.1001/jamanetworkopen.2025.8942 (PMC12059974; doi:10.1001/jamanetworkopen.2025.8942)
Supplement: Supplement 2. — Data Sharing Statement [file jamanetwopen-e258942-s002.pdf]

Data Sharing Statement

Zhang. Vehicle Age and Driver Assistance Technologies in Fatal Crashes Involving Teen and Middle-Aged Drivers. *JAMA Netw Open*. Published May 07, 2025.
doi:10.1001/jamanetworkopen.2025.8942

Data
Data available: Yes
Data types: Data (not involving human participants)
How to access data: Data used for this study are publicly available and can be downloaded from Fatality Analysis Reporting System's website. https://www.nhtsa.gov/research-data/fatality-analysis-reporting-system-fars
When available: beginning date: 11-01-2024

Supporting Documents
Document types: None

Additional Information
Who can access the data: N/A
Types of analyses: N/A
Mechanisms of data availability: N/A
